# Laminar Localization and Projection-Specific Properties of Presubicular Neurons Targeting the Lateral Mammillary Nucleus, Thalamus, or Medial Entorhinal Cortex

**DOI:** 10.1523/ENEURO.0370-16.2017

**Published:** 2017-05-15

**Authors:** Li-Wen Huang, Jean Simonnet, Mérie Nassar, Louis Richevaux, Roxanne Lofredi, Desdemona Fricker

**Affiliations:** 1 Inserm U1127, CNRS UMR7225, UPMC Université Paris 6 UMR S1127, Institut du Cerveau et de la Moelle Epinière, Sorbonne Universités, Paris 75013, France; 2 CNRS UMR 8119, Université Paris Descartes, France

**Keywords:** cell morphology, electrical properties, head direction, patch clamp, postsubiculum, retrograde tracing

## Abstract

The presubiculum (PrS) is part of an interconnected network of distributed brain regions where individual neurons signal the animals heading direction. PrS sends axons to medial entorhinal cortex (MEC), it is reciprocally connected with anterior thalamic nuclei (ATNs), and it sends feedback projections to the lateral mammillary nucleus (LMN), involved in generating the head direction signal. The intrinsic properties of projecting neurons will influence the pathway-specific transmission of activity. Here, we used projection-specific labeling of presubicular neurons to identify MEC-, LMN-, and ATN-projecting neurons in mice. MEC-projecting neurons located in superficial layers II/III were mostly regular spiking pyramidal neurons, and we also identified a Martinotti-type GABAergic neuron. The cell bodies of LMN-projecting neurons were located in a well-delimited area in the middle portion of the PrS, which corresponds to layer IV. The physiology of LMN projecting, pyramidal neurons stood out with a tendency to fire in bursts of action potentials (APs) with rapid onset. These properties may be uniquely adapted to reliably transmit visual landmark information with short latency to upstream LMN. Neurons projecting to ATN were located in layers V/VI, and they were mostly regular spiking pyramidal neurons. Unsupervised cluster analysis of intrinsic properties suggested distinct physiological features for the different categories of projection neurons, with some similarities between MEC- and ATN-projecting neurons. Projection-specific subpopulations may serve separate functions in the PrS and may be engaged differently in transmitting head direction related information.

## Visual Abstract

**Figure:**
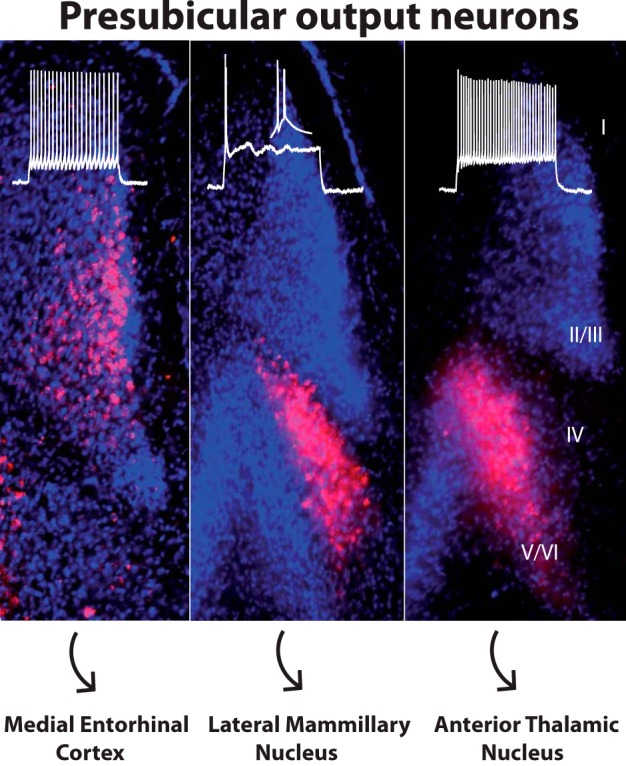

## Significance Statement

The presubiculum (PrS) is part of a brain wide network of head direction cells. It contributes to the generation of grid cell activity in the downstream medial entorhinal cortex (MEC), and it also feeds back information to subcortical input regions. Here, we identify projection-specific subpopulations of presubicular neurons. We show how they differ in their morphology, laminar location and in their electrophysiological tuning. Distinct presubicular cell types may provide specific coding capacities for distinct output channels of PrS.

## Introduction

Spatial navigation relies on extended brain circuits, including the hippocampal and parahippocampal network that support grid cell (Hafting et al., 2005), place cell (O'Keefe and Nadel, 1978), and head direction cell firing (Taube, 2007). The head directional signal first appears subcortically, in the reciprocally connected dorsal tegmental nucleus and lateral mammillary nucleus (LMN; Bassett et al., 2007; Clark and Taube, 2012). Vestibular sensory information crucially contributes to its generation (Stackman and Taube, 1997; Yoder and Taube, 2014). The head direction signal is then conveyed sequentially to the anterior thalamic nuclei (ATNs; Blair et al., 1998) and the dorsal part of the presubiculum (PrS; also termed postsubiculum; van Groen and Wyss, 1990a; Goodridge and Taube, 1997; Peyrache et al., 2015). In addition to thalamic head direction inputs, the PrS also receives inputs from visual cortex and retrosplenial cortex (Vogt and Miller, 1983; van Groen and Wyss, 1990a; Jones and Witter, 2007; Sugar and Witter, 2016). Self-motion and visual cues continually update the head direction signal (Taube, 2007), and when available, visual landmarks control the preferred firing direction (Zugaro et al., 2003).

It has been shown recently that the transmission of the head direction signal from the ATN is necessary for the generation and function of the grid cell activity in the medial entorhinal cortex (MEC; Winter et al., 2015). The presubicular projection may be essential for serially transferring the head direction signal from the ATN to the medial entorhinal grid cell system (Rowland et al., 2013; Preston-Ferrer et al., 2016). Other known projection targets of the PrS include feedback projections to the thalamus (van Groen and Wyss, 1990a,b; Ishizuka, 2001) and LMN (Allen and Hopkins, 1989; Gonzalo-Ruiz et al., 1992). Neurons of the presubicular microcircuit projecting to these two subcortical areas, ATN and LMN, have been identified as two nonoverlapping populations (Yoder and Taube, 2011). While head direction cells have been recorded *in vivo* across superficial and deep layers of PrS (Boccara et al., 2010; Tukker et al., 2015; Preston-Ferrer et al., 2016), the physiologic properties of presubicular cells that project to key areas of the head direction system, namely the upstream LMN and ATN, and the downstream MEC, have remained unclear.

In this study, we examine the morphology and physiology of retrogradely labeled presubicular neurons projecting to MEC, ATN, and LMN. We identify parameters that may have a significant influence on the function of presubicular efferent neurons. Principal component analysis (PCA) suggests a distinct profile of intrinsic properties of presubicular LMN-projecting neurons.

## Materials and Methods

### Animals

Experiments were performed on 22 male and female young adult C57BL/6 wild-type mice (postnatal day P29-P35 for stereotaxic injection and P31-P40 for recording), as well as six GAD67-GFP mice and two Sst^CRE^::tdTomato mice (Nassar et al., 2015). Animal care and use conformed to the European Communities Council Directive of 2010 (2010/63/EU) and French law (87/848). Our study was approved by the local ethics committee Charles Darwin N°5.

### Stereotaxic injections

Retrograde fluorescent tracers (Retrobeads, Lumafluor) were injected unilaterally into MEC, LMN, or ATN. Stereotaxic coordinates were: MEC, −4.65, 3.08, −4 mm; LMN, −2.8, 0.75, −5.35 mm; ATN, −0.8, 0.75, −3.2 mm (anteroposterior, mediolateral, dorsoventral to bregma). The procedure for injections followed a standard protocol (Mathon et al., 2015). Briefly, mice were deeply anesthetized with a mixture of ketamine and xylazine (80–20 mg/kg). A total of 150–300 nl of Retrobeads were injected with a Hamilton syringe at a speed of 40–60 nl/min. The animals were allowed to recover for at least 48 h.

### Slice electrophysiology

Horizontal slices (300–320 µm) containing PrS were prepared from mice that had been previously injected with Retrobeads. The cutting solution contained 125 mM NaCl, 25 mM sucrose, 2.5 mM KCl, 1.25 mM NaH_2_PO_4_, 25 mM NaHCO_3_, 2.5 mM glucose, 0.5 mM CaCl_2_, and 7 mM MgCl_2_ (cooled to 2–6°C, bubbled with 95% O_2_/5% CO_2_). Slices were incubated for 15 min at 36°C in a holding chamber with aCSF composed of 124 mM NaCl, 2.5 mM KCl, 10 mM NaH_2_PO_4_, 26 mM NaHCO_3_, 11 mM glucose, 2 mM CaCl_2_, and 2 mM MgCl_2_ (bubbled with 95% O_2_/5% CO_2_). After incubation, slices were stored at room temperature. For whole-cell recordings, slices were bathed in carbogenated aCSF at 32-34°C. Retrobeads containing neurons of PrS were identified under a Axioscope 2FS plus microscope (Zeiss), equipped with appropriate LED illumination (Cairn). Pipettes were pulled into patch electrodes with 4- to 6-MΩ resistance and filled with a potassium-based intracellular solution, pH 7.3, composed of 140 mM K-gluconate, 1.2 mM KCl, 10 mM HEPES, 0.2 mM EGTA, 2 mM MgCl_2_, 4 mM MgATP·2H_2_O, 0.4 mM Na_3_GTP·2H_2_O, and 10 mM Na phosphocreatine. Biocytin (3 mg/ml) was added for *post hoc* revelation of cellular morphology. Pipette capacitance was compensated. Electrophysiological signals were sampled at 50 kHz and filtered at 5-6 kHz (Multiclamp 700B or Axopatch 200A amplifier, Molecular Devices) in whole-cell current-clamp mode. Data acquisition and analysis were performed, respectively, in pClamp (Molecular Devices), Axograph, and MATLAB.

The resting membrane potential was determined in voltage-follower mode shortly after breaking in and averaging the membrane potential over 20 s. During the step current injection protocol, baseline membrane potential was maintained at −65 mV. Membrane responses in current clamp were elicited by injecting hyperpolarizing to depolarizing current steps of 800-ms duration (typically starting at −300 pA; 10–20 pA increments). The membrane voltage was plotted as a function of current commands, and a linear fit was created between −70 and −60 mV, the slope of which indicated input resistance, R_input_. The membrane time constant, tau, was calculated by fitting a double exponential function to an averaged membrane response to a small negative current (potential difference ΔV < 10 mV; Golowasch et al., 2009). The shorter of the two time constants was used. Sag ratio was measured by averaging the ratio ΔV_min_/ΔV_steady-state_ of three data points around −100 mV (ΔV_min_ = V_min_ − V_baseline_; Δ_steady-state_ = V_steady-state_ − V_baseline_).

The properties of the first action potential (AP) at rheobase (the minimal current required to discharge APs) were characterized: AP threshold (a point at the foot of the AP where dV/dt > 30 V/s), AP amplitude (from threshold to peak), AP width (width at half-maximum amplitude), afterhyperpolarization amplitude (AHP, from threshold to the trough of the AHP), and AP maximum depolarization and repolarization rates. The latency of the first AP was measured from the onset of the rheobase current step to the peak of the AP. The firing rate at twice rheobase was determined. The current step that gave the AP firing rate closest to 15 Hz was analyzed for bursting behavior of spiking, quantified by fast-doublet index (the averaged interspike interval over the first interval, only applicable for neurons that fired more than two spikes during positive current steps). Firing rate increases were quantified by calculating the initial (four to five current points from rheobase) slope of the frequency-current relationship (f-I slope).

### Histology and anatomy

Slices were fixed in 4% paraformaldehyde in 0.1 M phosphate buffer (PB) overnight, then incubated with a blocking solution (2% milk in PB supplemented with 0.3–1% Triton X-100). Streptavidin-Cy3 or Cy5 conjugate (1:500; Life Technologies) was used for biocytin staining, and DAPI (1:1000) to stain nuclei. Sections were imaged using a pseudo-confocal Olympus IX81 microscope, and Volocity software for analysis, or a Zeiss LSM 710 confocal microscope. Retrobeads-labeled MEC-projecting neurons of the PrS were visualized in stacks of confocal images of NeuN-stained 60-µm sections and counted manually, in four ipsilateral and three contralateral sections from two mice. Their laminar distribution was quantified in each section (total 100% per section). Retrobeads labeled GABAergic neurons were counted in three sections from one GAD67-GFP mouse and from one SstCre::tdTomato mouse. The Neurolucida software was used for 3-D computer-aided morphologic reconstruction of biocytin-filled neurons as in (Simonnet et al., 2013).

### Statistical analysis

The PCA and Ward’s unsupervised cluster analysis (Ward, 1963; Simonnet et al., 2013) of presubicular projection neurons was implemented using MATLAB, and based on 11 electrophysiological parameters (Table 1): resting membrane potential, input resistance, tau, sag ratio, AP threshold, AP amplitude, AP width, AP AHP, AP latency, firing rate at double rheobase, f-I slope. Measurements for each variable x_i_ in our data set were transformed to standard scores across all cells based on the formula ([(x_i_ – mean)/SD]; Romesburg, 1984). To avoid artificially weighting highly correlated variables, the AP maximum depolarization rate and repolarization rate were not included, because they were highly correlated with AP amplitude and AP width, respectively (*r* > 0.8 in the correlation matrix), and their value was lower in the PC loadings table (Tsiola et al., 2003).

**Table 1.** Intrinsic properties of projection-specific presubicular neurons

	MEC projectors			LMN projectors			ATN projectors			MEC proj.	
	Mean	SEM	*N*	Mean	SEM	*N*	Mean	SEM	*N*	Martinotti	*N*
**Resting membrane potential (mV)**	−70	2	18	−62	2	18	−68	2	20	−33	1
**Input resistance (MΩ)**	367	49	18	166	17	18	444	30	20	262	1
**Time constant tau (ms)**	18	1	18	13	1	18	21	1	20	11	1
**Sag ratio**	1.06	0.01	18	1.22	0.04	18	1.18	0.04	20	1.16	1
**AP threshold (mV)**	−33	1	18	−37	1	18	−33	1	20	−32	1
**AP amplitude (mV)**	80	1	18	81	3	18	76	1	20	84	1
**AP width (ms)**	0.63	0.02	18	0.53	0.02	18	0.61	0.03	20	0.34	1
**AP AHP (mV)**	−17.0	0.7	18	−8.1	1.1	18	−17.4	0.5	20	−26.8	1
AP max. depol. (V/s)	457	25	18	524	38	18	417	21	20	537	1
AP max. repol. (V/s)	−119	4	18	−144	6	18	−126	8	20	−259	1
**Latency to spike**	146	25	18	48	5	18	281	62	20	74	1
**Firing rate at double rheobase (Hz)**	25	1	18	13	3	18	28	4	20	26	1
Fast doublet index	1.7	0.1	18	10.1	2.4	11	1.7	0.2	20	2.4	1
**f-I slope (Hz/nA)**	334	34.8	18	190	37	18	499	54	20	654	1

The parameters in bold are used for PCA and cluster analysis in Figure 4.

## Results

### Anatomic segregation of LMN, ATN, and MEC projectors in the PrS

**Figure 1.**
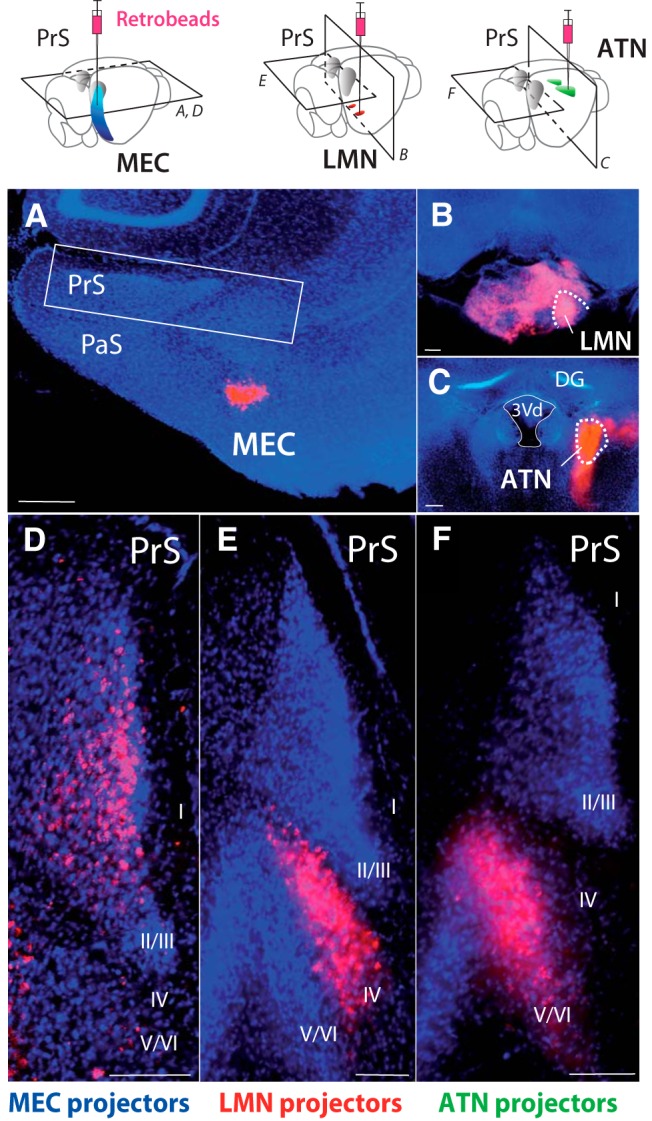
Anatomic segregation of presubicular neurons that project to MEC, LMN, or ATN. ***A***, Injection of retrobeads into layer III of MEC. Low-magnification image of a horizontal section of the temporal lobe. ***B***, Injection of retrobeads into LMN (coronal section). ***C***, Injection of retrobeads into ATN (coronal section). ***D***, Higher-magnification image of the PrS (rectangle in ***A***). Retrogradely labeled MEC-projecting neurons are mostly found in superficial layers of PrS. ***E***, LMN-projecting neurons are confined to layer IV of PrS (horizontal section, same animal as in ***B***). ***F***, ATN-projecting neurons are present in layers V-VI of PrS (horizontal section, same animal as in ***C***). Retrobeads in red, DAPI staining in blue. PaS, parasubiculum; DG, dentate gyrus; 3Vd, dorsal third ventricle. Scale bars, 200 µm (***A****–****C***) and 100 µm (***D****–****F***).

Retrograde fluorescent tracer was injected in MEC (10 mice), LMN (9 mice), or ATN (7 mice), to identify presubicular projection neurons. Injection sites were confirmed *post hoc* in horizontal sections for MEC (Fig. 1*A*), and in coronal sections for LMN (Fig. 1*B*) and ATN (Fig. 1*C*). MEC injection targeted the superficial layers of the MEC, and in some cases deep layers were also hit. Cases where MEC injections were not entirely restricted to the MEC were excluded from analysis. LMN injection sites and ATN injection sites were confirmed in three cases by slicing the rostral half of the brain in the coronal plane, while the distribution of retrogradely labeled neurons was observed in horizontal sections of PrS, contained in the caudal half of the brain (Fig. 1, illustrations).

Retrobeads injection into MEC resulted in bilateral retrograde labeling in the PrS. Both ipsi- and contralaterally, labeled neurons were most numerous in presubicular layer III, some were located in layer II, and some in deep layers (Fig. 1*D*). Retrobeads-labeled neuron counts resulted in highest numbers in layer III (ipsi, 686 cells, 55 ± 8%; contra, 258 cells, 57 ± 4%), and in layer II (ipsi, 213 cells, 33 ± 7%; contra, 116 cells, 41 ± 7%). A total of 50 cells (6 ± 2%) were Retrobeads-labeled in ipsilateral layer IV and 23 cells in contralateral layer IV (2 ± 2%), 41 cells (6 ± 4%) in ipsilateral layer V/VI, and 9 cells (1 ± 1%) in contralateral layer V/VI (total ipsilateral, 990 cells in four slices; total contralateral, 406 cells in three slices). Retrobeads-labeled neuron somata were also observed in other brain regions known to project to the MEC, including the parasubiculum, subiculum, CA1 and postrhinal cortex, which underscores the specificity and efficacy of our retrograde tracing. Following Retrobeads injection into LMN, labeled neurons were located in the ipsilateral PrS, in a well-defined area in the center portion of presubicular layers. This area corresponded to the cytoarchitectonic limits of layer IV (Fig. 1*E*). No beads-labeled neuron somata were observed in layers I–III, nor in V/VI. Following ATN injection, Retrobeads-labeled neurons were found in the deep portion of the ipsilateral PrS, in layers V/VI (Fig. 1*F*), but not in superficial layers I-III nor in layer IV. The home layers of LMN- and ATN-projecting neurons were closely adjacent and we consider the labeling of somata of these two projecting neuron populations as defining the limits of presubicular layers IV versus V/VI, respectively (Yoder and Taube, 2011).

### Morphology and intrinsic electrophysiological properties of presubicular projecting neurons

**Figure 2.**
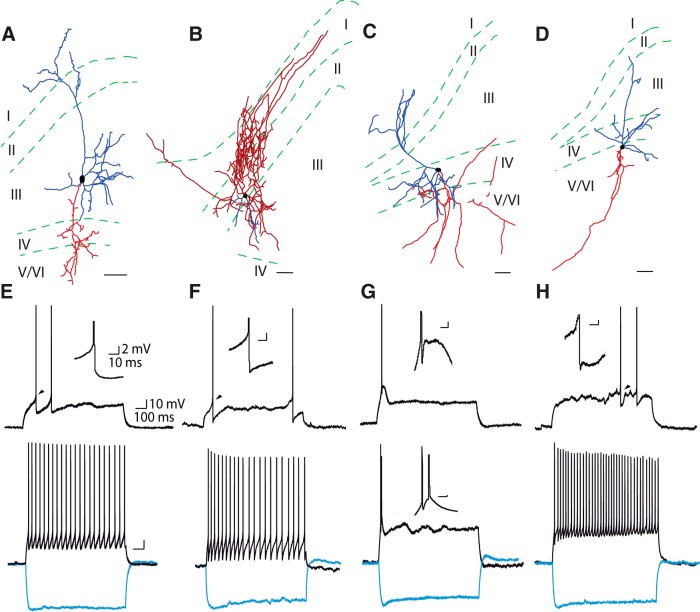
Morphology and firing patterns of retrogradely labeled presubicular projecting neurons. ***A***, ***E***, MEC-projecting pyramidal neuron. ***B***, ***F***, MEC-projecting Martinotti interneuron. ***C***, ***G***, LMN-projecting neuron. ***D***, ***H***, ATN-projecting neuron. ***A–D***, Reconstruction of cell morphology with dendrites in blue and axons in red. Scale bars, 50 µm. Additional examples of reconstructions of presubicular projection neurons can be found in Extended Data Fig. 2-1. ***E–H***, Firing patterns at rheobase (upper traces) and at double rheobase (lower traces). Membrane voltage responses to hyperpolarizing current steps of -150 pA are shown in light blue. Insets show larger scale traces of the first AP.

**Figure 3.**
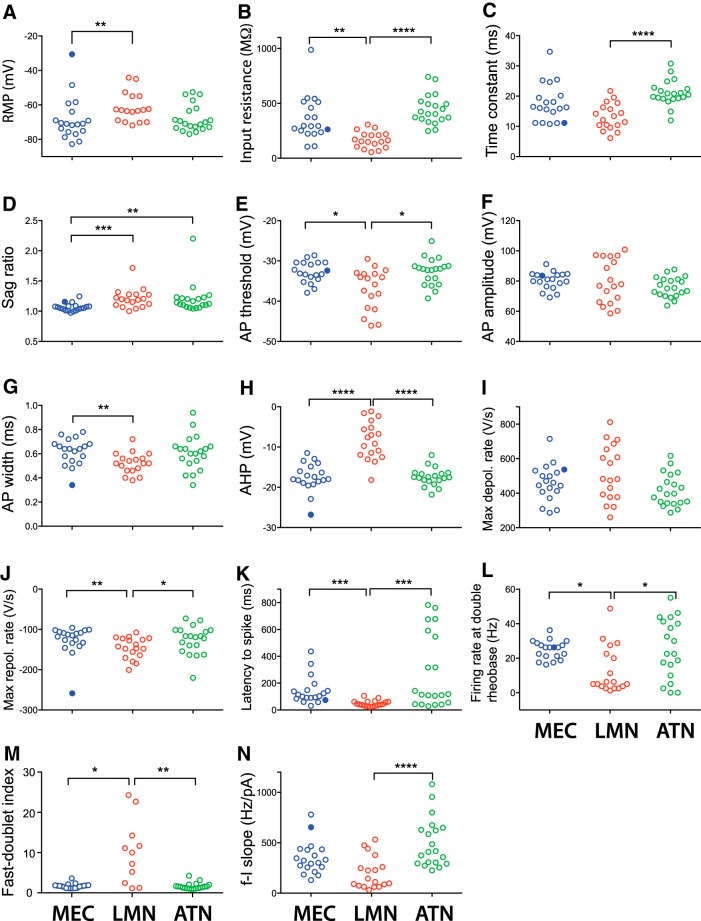
Comparison of intrinsic properties of presubicular neurons that project to MEC (blue), LMN (red), or ATN (green). ***A***, Resting membrane potential (RMP). ***B***, Input resistance. ***C***, Time constant. ***D***, Sag ratio. ***E***, AP threshold. ***F***, AP amplitude. ***G***, AP width. ***H***, AHP. ***I***, AP maximum depolarization rate. ***J***, AP maximum repolarization rate. ***K***, Latency to first spike at rheobase. ***L***, Firing rate at double rheobase. ***M***, Fast doublet index. ***N***, f-I slope. Kruskal Wallis and Dunn's multiple comparison *post hoc* test were performed for significance among projecting neurons, *p < 0.05, **p < 0.01, ***p < 0.001, ****p < 0.0001. Data for one MEC projecting Martinotti-type interneuron are represented as filled blue circles in the graphs, but they are not included for the statistical comparison among the groups of MEC-, LMN-, or ATN-projecting neurons.

Retrogradely labeled presubicular neurons projecting to MEC, LMN, or ATN were targeted for *in vitro* whole-cell recordings. The injection sites into MEC or ATN were routinely checked in horizontal sections in all animals used for electrophysiology. LMN was not contained in horizontal slices, but, following our results from anatomic analysis, LMN injections were estimated to be correct, if labeled neurons were present specifically and exclusively in layer IV of PrS. Active spiking behavior and passive membrane properties of 57 projecting neurons were measured and quantified. Table 1 gives an overview of mean ± SEM of all measured parameters. Figure 2 shows an illustrative example of morphology and electrophysiology for each group of projecting neurons (more examples are included as Extended Data, Fig. 2-1). Graphs in Figure 3 give all data points for all parameters, and statistical comparisons between the different groups of projecting neurons.

10.1523/ENEURO.0370-16.2017.f2-1 Figure 2-1 Reconstructions of 23 biocytin filled, retrogradely labeled presubicular principal neurons. **A**, MEC-projecting neurons. **B**, LMN-projecting neurons. **C**, ATN-projecting neurons. Dendrites in blue and axons in red. Presubicular layers II and IV indicated by parallel black lines. Scale bars, 100µm. PaS, Parasubiculum; Sub, Subiculum. Download Figure 2-1, EPS file.

### MEC projectors in superficial layers: regular spiking pyramidal neurons or interneurons

We recorded from 19 retrogradely labeled presubicular MEC-projecting neurons, which were all located in superficial layers. A total of 5/19 were located close to the border of layer II/III, the other 14/19 in layer III. No deep layer MEC-projecting neurons were recorded. Reconstruction of dendritic and axonal morphologies of recorded and biocytin filled MEC-projecting neurons revealed that they were mostly typical layer III pyramidal neurons, with apical dendrites arborizing in layer I, basal dendrites in layer III, and axons branching across deep layers. A total of 90% of mean dendritic length was distributed across layers I–III (Figs. 2*A*, 2-1; Tables 2, 3). Interestingly, one Retrobeads-labeled MEC-projecting neuron in layer III was nonpyramidal and identified as a putative long-range projecting GABAergic neuron with a typical Martinotti type morphology (Fig. 2*B*). The axon of the Martinotti cell arborized densely in superficial layers and its dendrites extended across deep layers.

**Table 2.** Dendritic layer length of projection-specific presubicular neurons

	MEC projectors			LMN projectors			ATN projectors		
	Mean	SEM	*N*	Mean	SEM	N	Mean	SEM	*N*
Layer I (µm)	447	163	8	319	122	8	24	17	7
Layer II (µm)	126	38	8	75	16	8	54	27	7
Layer III (µm)	1144	246	8	407	77	8	174	66	7
Layer IV (µm)	144	98	8	802	134	8	433	206	7
Layer V/VI (µm)	2	2	8	616	134	8	617	131	7
Extra PrS (µm)	50	42	8	196	106	8	340	245	7
**Total (µm)**	1911	335	8	2415	266	8	1643	354	7

Figure 2-1 shows corresponding biocytin reconstructions of projection neurons.

**Table 3.** Axonal layer length of projection-specific presubicular neurons

	MEC projectors			LMN projectors			ATN projectors		
	Mean	SEM	*N*	Mean	SEM	*N*	Mean	SEM	*N*
Layer I (µm)	0	0	8	0	0	8	0	0	7
Layer II (µm)	10	10	8	0	0	8	0	0	7
Layer III (µm)	213	67	8	77	71	8	0	0	7
Layer IV (µm)	157	78	8	333	136	8	0	0	7
Layer V/VI (µm)	223	113	8	506	166	8	248	60	7
Extra PrS (µm)	296	294	8	215	180	8	477	109	7
**Total (µm)**	899	400	8	1131	295	8	725	125	7

Figure 2-1 shows corresponding biocytin reconstructions of projection neurons.

Pyramidal MEC-projecting neurons had a mean resting membrane potential of −70 ± 2 mV (*n* = 18). Following positive current injections, they fired at high frequencies with little adaptation (*n* = 18; Fig. 2*E*). The firing frequency at double rheobase current in pyramidal MEC projectors was 25 ± 1 Hz, significantly higher than in LMN projectors (13 ± 3 Hz; *p* < 0.05; Fig. 3*F*). Negative current injections revealed very little voltage sag. The sag ratio was 1.06 ± 0.01, significantly smaller than in LMN or ATN projectors (*p* < 0.001 and *p* < 0.01, respectively; Fig. 3*D*). The latency to the first spike for rheobase current injections was 146 ± 25 ms, significantly longer than for LMN projectors, but not significantly different from ATN projectors (*p* < 0.001 and n.s., respectively; Fig. 3; Table 1).

Voltage recordings of the MEC projecting Martinotti type interneuron are shown in Figure 2*F*. Its intrinsic properties are given in Table 1, and data points appear as filled blue circles in the graphs in Figure 3. We observed spontaneous AP firing from a depolarized membrane potential (average membrane potential, −33 mV) with prominent AP AHPs. The AHP amplitude was −26.8 mV in this MEC projecting Martinotti cell, deeper than the average AHP in pyramidal MEC-projecting neurons (−17.0 ± 0.7 mV; Fig. 3*H*). Its sag ratio was 1.16, slightly higher than average for the pyramidal MEC-projecting neurons. The Martinotti cell AP width was 0.34 ms, well below the width of pyramidal MEC-projecting neurons (0.63 ± 0.02 ms; Fig. 3*G*). AP threshold, amplitude and firing rate at double rheobase were close to average pyramidal MEC projectors (Fig. 3*E*,*F*,*L*).

To examine whether presubicular GABAergic neurons regularly participate in the projection to MEC, Retrobeads were injected in MEC in transgenic mice where all GABAergic neurons (GAD67-GFP line) or a subpopulation of somatostatin-expressing neurons (SstCre::tdTomato line) can be identified by their green or red fluorescence. We found that six GABAergic presubicular neurons in one GAD67-GFP mouse and six presubicular tomato-expressing neurons in one SstCre::tdTomato mouse also contained Retrobeads. Overall, <1% of MEC-projecting neurons were GABAergic. Retrobeads-labeled GABAergic neurons are thus a small minority of presubicular MEC-projecting neurons. Due to small sample size, we did not include the unique recording of a MEC projecting Martinotti cell in subsequent PCA or cluster analysis.

### LMN projectors: bursting pyramidal neurons in layer IV

A total of 18 presubicular LMN-projecting neurons were recorded and biocytin filled. All were layer IV neurons with pyramidal shape, an apical dendrite arborizing in layer I (except for one inverted pyramid; Fig. 2-1), basal dendrites mainly in layer IV and V/VI (58% of mean dendritic length), and their axon branching across deep layers (Figs. 2*C*, 2-1; Tables 2, 3). The average resting membrane potential of LMN-projecting neurons was -62 ± 2 mV, significantly more depolarized than in the group of pyramidal MEC projectors (*p* < 0.01; Fig. 3*A*), and they had a low input resistance (166 ± 17 MΩ**;** *p* < 0.0001 compared with ATN projectors; Fig. 3*B*). Following positive current injections, LMN projectors tended to fire in an initial burst of two spikes with short latency (48 ± 5 ms, *n* = 18; Figs. 2*G*, 3*K*). The first spike at rheobase occurred at significantly shorter latency than in either MEC or ATN projectors (*p* < 0.001). Short latency AP firing in LMN projectors was favored by a short membrane time constant, significantly shorter than in ATN projectors (tau, 13 ± 1 ms; *p* < 0.0001; Fig. 3*C*). We also note the presence of a depolarizing current at the onset of a depolarizing step (Fig. 2*G*) promoting short latency firing of either bursts or single spikes. After the initial discharge, cells could fire regular sparse APs. A depolarizing envelope always underlay the first spike, and the amplitude of the AHP was low (−8.1 ± 1.1 mV, compared with −17.4 ± 0.5 mV for ATN or 17.0 ± 0.7 mV for pyramidal MEC-projecting neurons; *p* < 0.0001; Fig. 3*H*), evidence for the ability to fire bursts (Connors and Gutnick, 1990; Simonnet et al., 2013). The bursting tendency was quantified by the fast-doublet index, which was 10.1 ± 2.4 for LMN projectors (*n* = 11), while MEC projectors (*n* = 18) and ATN projectors (*n* = 20) both had very low average fast-doublet indices of 1.7 ± 0.1 and 1.7 ± 0.2 (Fig. 3*M*). Most LMN projectors displayed prominent sag during negative current steps, and rebound depolarization after the offset (Fig. 2*G*). The sag ratio was 1.21 ± 0.28 (*n* = 18), significantly higher than in pyramidal MEC projectors (*p* < 0.001; Fig. 3*D*).

### ATN projectors: regular firing pyramidal neurons in layer V/VI

A total of 20 presubicular ATN-projecting neurons were recorded. They were deep layer neurons with pyramidal shape, as the example in Figure 2*D*. Dendrites distributed across all layers, with 69% of mean dendritic length in layers IV and V/VI. The axons ran through deep layers V/VI to exit PrS (Figs. 2*D*, 2-1; Tables 2, 3). The mean resting membrane potential of ATN-projecting neurons was −68 ± 2 mV (*n* = 20; Fig. 2*H*), similar to MEC-projecting neurons. Values for mean input resistance (444 ± 30 MΩ) and membrane time constant (21 ± 1 ms) were high, and both parameters were significantly higher than in LMN projectors (*p* < 0.0001; Fig. 3*B*,*C*). The latency to spike was long at rheobase in some cells, but not in all cells (average late ncy, 281 ± 62 ms). For larger current injections, ATN projectors fired with little adaptation (*n* = 20; Fig. 2*H*). For negative current injections, ATN-projecting neurons showed a large sag (sag ratio, 1.18 ± 0.04; Fig. 3*D*), which appeared somewhat slower compared with LMN-projecting neurons (Fig. 2*G*,*H*). The AP width in ATN projectors was 0.61 ± 0.03 ms, with a slower maximum repolarization rate compared with LMN projectors (*p* < 0.05; Fig. 3*J*).

### Physiologic segregation of projection-specific presubicular neurons

**Figure 4.**
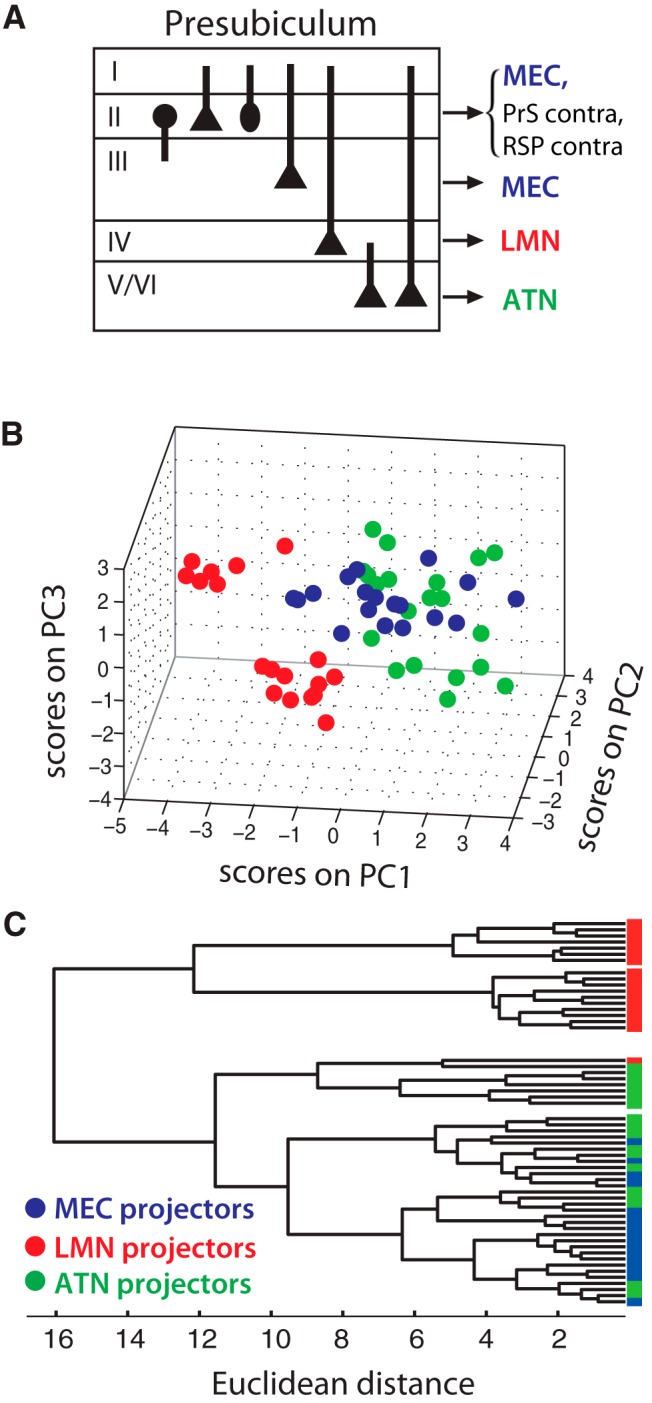
Segregation of MEC projectors (blue), LMN projectors (red) and ATN projectors (green) in PrS. ***A***, Schematic of layering and cell types of the PrS with their preferential projection profiles. Layer II contains neurons targeting the MEC, contralateral PrS, and retrosplenial cortex (Preston-Ferrer et al., 2016). Layer III contains mostly pyramidal MEC projectors. Large pyramidal neurons targeting LMN lie in layer IV. Neurons targeting ATN lie in layer V/VI; their dendrites may or may not reach to layer I (Fig. 2-1) ***B***, Segregation of projection-specific presubicular neurons based on electrophysiological parameters. Score plot of projecting neurons on PC1, PC2, and PC3 planes. ***C***, Cluster analysis of presubicular projecting neurons.

We next wished to examine the range of intrinsic properties of presubicular projection neurons (Fig. 4*A*) using PCA. PCA selected variables that contributed most to the overall variability and, thus, were most important in distinguishing different physiologic cell groups. The first PC (PC1) captured most of the variability (34%). The parameters that were positively correlated with PC1 included passive properties, tau and input resistance, and active properties, f-I slope, firing rate at double rheobase, AP width, AP threshold, latency to spike. PC2 mostly correlated with sag ratio, f-I slope, firing rate at double rheobase, and PC3, mostly AP width, capturing an additional 15.9% and 14.7% of the total variability, respectively. Thus, ∼64.6% of the total variability could be explained by the first three PCs. PCA revealed a separation between LMN-projecting neurons (Fig. 4*B*, red dots) and ATN/MEC-projecting neurons (Fig. 4*B*, green dots/blue dots). The same data set was then submitted to unsupervised cluster analysis. The projection–specific subpopulations of neurons are reflected in the dendrogram as two major clusters, separating LMN projectors (Fig. 4*C*, red) from MEC projectors (Fig. 4*C*, blue) and ATN projectors (Fig. 4*C*, green). The two subclusters in the LMN projecting group corresponded to two subpopulations in PCA as well. ATN-projecting neurons were separated into two different subclusters with one subcluster sharing some similarity with MEC-projecting neurons.

## Discussion

We have examined projection-specific subpopulations of presubicular neurons in the mouse. MEC, LMN, and ATN-projecting neurons largely segregate anatomically in their layer distribution and also physiologically in their intrinsic properties. Presubicular LMN projectors form a population with unique properties: they are pyramidal neurons located in layer IV, characterized by intrinsic bursting discharge behavior and short latency firing. Presubicular ATN-projecting neurons are found in deep layers V/VI. They are anatomically apart from LMN projectors, and their regular spiking firing pattern also distinguishes them from LMN projectors. The large majority of MEC-projecting neurons are located in superficial layers II and III of the PrS, a population distinct from LMN- or ATN-projecting neurons. Superficial pyramidal cell MEC projectors have a regular spiking firing behavior and are relatively similar to ATN-projecting neurons. A few MEC projectors are in layers IV and V/VI, and those may or may not overlap with LMN- or ATN-projecting neurons. Our results show distinct electrophysiological tuning of presubicular output neurons allowing for pathway-specific transmission of head direction related information.

The three main projection-specific classes of presubicular neurons examined here fit well with a previous classification of presubicular neurons in rats, where neurons were classified based on cellular and morphologic criteria (Simonnet et al., 2013). That study had distinguished regular-firing, small pyramidal neurons in layer II/III of PrS, intrinsically bursting, large pyramidal cells with a prominent sag and rather depolarized membrane potential in layer IV, and heterogeneous regular-firing neurons in layer V/VI, with larger sags than superficial layer cells. The preserved cell layer structure and firing patterns across species, mouse and rat, point to an important functional role of these neuronal subpopulations in the rodent head direction circuit.

Retrograde tracing of MEC-projecting neurons labeled many somata in layer II/III of PrS and a few in deep layers. Our findings confirm previous tracing studies showing that pre- and postsubiculum project bilaterally to the entorhinal cortex (van Groen and Wyss, 1990b; Caballero-Bleda and Witter, 1993; van Haeften et al., 1997; Honda and Ishizuka, 2004; Rowland et al., 2013). Specifically, superficial layer III of PrS had been found to send a major axonal projection to ipsilateral MEC layer I/III (Caballero-Bleda and Witter, 1993; Honda and Ishizuka, 2004) and a minor projection to MEC layer II (Rowland et al., 2013). Some presubicular layer V pyramidal cells also send axons to ipsilateral entorhinal cortex, either to layer II and III of MEC (Honda et al., 2011) or to deep layers of MEC (Honda and Ishizuka, 2004). While our results are coherent with the existence of deep-layer presubicular MEC-projecting neurons, our physiologic analysis focused on the major projection originating from presubicular layers II/III.

Presubicular layer II and III can be distinguished based on differences in cell body densities, and the distance to the cell sparse layer I. Immunohistochemical labeling, such as calbindin staining, can also help to define layer II (Preston-Ferrer et al., 2016). In our data set of MEC-projecting neurons, most were located in layer III. A smaller portion was located close to the interface of layers II and III. *In vivo* recordings have identified presubicular pyramidal cells in layer III as head direction cells (Peyrache et al., 2015; Tukker et al., 2015; Preston-Ferrer et al., 2016), and their regular firing behavior with little adaptation seems well suited for transmitting directional information to MEC. Functionally, the PrS → MEC projection might be key for spatial information generation in the MEC. The nature of the target neurons in MEC remains to be elucidated: do presubicular head direction neurons contact grid cells, border cells or other head direction cells?

A few MEC-projecting neurons (<1%) were GABAergic, and those cells were strictly located in the ipsilateral PrS, in layer II or III. Previous reports had estimated 20-30% of MEC projection neurons to be GABAergic (van Haeften et al., 1997). This much higher percentage could be due to a species difference (mouse vs rat), or to a lesser uptake of Retrobeads by interneurons. Also, our presubicular slices were taken at a mid-dorsoventral level, while the GABAergic projection to MEC may be limited to the most dorsal part of the PrS (van Haeften et al., 1997). Presubicular GABAergic neurons may or may not be directionally tuned. Some GABAergic neurons, the fast spiking presubicular interneurons, are sensitive to angular head velocity (Preston-Ferrer et al., 2016). The specific role of inhibition through presubicular MEC projecting interneurons remains to be elucidated.

As noted above, PrS → LMN-projecting neurons were exclusively distributed in layer IV of the PrS and PrS → ATN-projecting neurons were only found in layer V/VI (Fig. 4*A*). We thus confirm for mice a previous report from rats, showing that these projection neurons constitute nonoverlapping populations in distinct presubicular layers (Yoder and Taube, 2011). In addition to this anatomic segregation of the two projection-specific subpopulations, we reported for the first time that LMN-projecting neurons and ATN-projecting neurons are also physiologically distinct: LMN-projecting neurons are burst-firing neurons with short latency to spike, while ATN-projecting neurons are regular-firing neurons.

We also find some physiologic diversity within presubicular efferent neuron populations. While the physiologic make-up of a neuronal population might never be entirely identical, the target areas of presubicular projections may not be entirely homogeneous either. Rodent mammillary body and anterior thalamus are subdivided brain structures. Adjacent to the LMN lies the lateral part of the medial mammillary nucleus, which also receives presubicular inputs (van Groen and Wyss, 1990b). It is possible that the two separated LMN-projecting groups in our PCA and clustering analysis correspond to presubicular neurons that contact either of these two subdivisions of the mammillary body. We also observed some diversity for thalamic projection neurons, and indeed, presubicular neurons may target several thalamic subnuclei. The presubicular projection to the thalamus reaches densely the anterodorsal nucleus, and to a lesser degree the anteroventral and laterodorsal nucleus (van Groen and Wyss, 1990a). Possibly the physiologic heterogeneity of presubicular thalamic projecting neurons relates to the heterogeneity of their thalamic target nuclei. An intersectional labeling approach of projection neurons in combination with molecular markers such as transcription factors could further narrow down a defined population of corticothalamic projection neurons (Sürmeli et al., 2015; Woodworth et al., 2016).

Presubicular LMN-projecting neurons may be the preferential route for visual information to update the subcortical head direction signal (Yoder and Taube, 2011; Yoder et al., 2015; Bicanski and Burgess, 2016). Visual cortex sends direct projections to the superficial layers of PrS (Vogt and Miller, 1983) and also indirectly via the retrosplenial cortex (Sugar and Witter, 2016). Presubicular layer IV pyramidal neurons could receive these inputs directly, given their prominent dendritic arborization that extends in superficial layers (Yoder and Taube, 2011; Simonnet et al., 2013). We suggest that the fast integrative properties and the intrinsic burst firing behavior of layer IV PrS → LMN-projecting neurons favor fast and reliable transfer of information. Indeed, the visual update of the head direction signal becomes effective within a very short latency (∼80 ms) in thalamus (Zugaro et al., 2003), suggesting that the synaptic transmission from visual cortex to ATN should be very fast, either via PrS → LMN → ATN or directly via the PrS → ATN projection. Efficient excitatory drive might be rapidly sent in parallel pathways, to the LMN, and to neurons projecting to ATN, providing convergent information from the PrS and LMN to update the head direction signal in thalamus.

While presubicular head direction cells signal the current head direction, LMN (Stackman and Taube, 1998) and ATN (Goodridge and Taube, 1997) head direction cells anticipate future head direction. The anticipatory time interval depends on the frequency of visual updates: feedback should act intermittently rather than continuously and, modeling studies suggest, visual update should best be delivered at low frequencies (<1 Hz; van der Meer et al., 2007). Single spike or intrinsically burst firing of presubicular LMN projecting layer IV neurons may provide the necessary sparse coding.

While *in vivo* head direction cells were recorded across superficial and deep layers of PrS (Boccara et al., 2010), we revealed for the first time different physiologic signatures of presubicular cells that project to key areas of the head direction system, namely the upstream LMN and ATN, and the downstream MEC. The presubicular projection to the LMN relays visual information to the head direction system and may enhance head direction signal stability and accuracy (Yoder et al., 2015). The bursting nature of PrS → LMN-projecting neurons ensures that visual feedback is sent sparsely but efficiently with little delay to the subcortical generating circuit of the head direction signal.

## Synthesis

Reviewing Editor: Marlene Bartos, University of Freiburg Decisions are customarily a result of the Reviewing Editor and the peer reviewers coming together and discussing their recommendations until a consensus is reached. When revisions are invited, a fact-based synthesis statement explaining their decision and outlining what is needed to prepare a revision will be listed below. The following reviewer(s) agreed to reveal their identity: Imre Vida

The present manuscript focuses on the neuronal subpopulations of the presubiculum with distinct projections to the entorhinal cortex, the lateral mammillary nucleus and the anterior thalamic nucleus. The authors show how they form non-overlapping subsets which differ in their laminar localization and in their passive and active intrinsic electrophysiological properties. These results suggest that distinct presubicular cell types provide specific coding capacities for the diverse output streams of presubiculum.

The study is based on a focused set of experiments which combine retrograde labeling, in vitro electrophysiology and morphological analysis. The results are convincing and fully support the conclusions. While the results and conclusions are not entirely novel, by providing a combined analysis of the target region, laminar localization, anatomical and physiological properties of the neurons the manuscript constitutes a unique contribution to the still rather sparse literature on the cellular organization of the presubiculum. In view of the central position of this area in the spatial navigation system of the rodent brain, these results are of interest to a broader neuroscience audience.

A major weakness of the study is the limited scope of the anatomical, morphological analysis. The authors have a sample of about 18 - 20 neurons for each projection type, but provide only a single “representative” reconstruction for each of these in Figure 1. This data set is sufficiently large to analyze main morphological features of the neurons, which define integrative properties of the neurons, and to demonstrate how homogeneous or heterogeneous these neurons are.

A further shortcoming of the study is the lack of characterization of layer II neurons which are known to preferentially project to the contralateral presubiculum or the retrosplenial cortex (Preston-Ferrer et al., 2016).

Specific points

Title

The title does not mention the largely non-overlapping laminar localization of the neurons; while this is not a genuinely novel finding, it is a major aspect of the study and can also explain the distinct morphological and physiological properties.

Abstract

- Please check whether to capitalize (or not) the names of the nuclei: “anterior thalamic nuclei” vs. “Lateral Mammillary Nucleus”.

- “which defines layer IV” - Do you mean “corresponds to layer IV”?

- Please indicate explicitly that LMN projecting and ATN projecting neurons were also pyramidal cells. This is currently not clear from the abstract.

Methods

Histology and anatomy: “Slices were fixed in 4% paraformaldehyde” - please indicate the vehicle solution (0.1 M PB?).

Results, Page 6, middle:

Regarding the bilateral retrograde labeling of the presubiculum from the MEC could you please compare the extent/strength of the labeling for the two sides?

For the quantification of the laminar distribution of the retrogradely labeled neurons, could you please provide not only the absolute numbers, but also the percentages?

Page 8, middle:

For the MEC projecting neurons it is indicated what the proportion of the identified GABAergic neurons. Could you please also give the proportion of the projection neurons within the identified Somatostatin expressing neuron population in the SstCre::tdTomato line?

Figure 4 provides a good visual overview of the man findings but stops short of providing a comprehensive summary of current knowledge. A schematic of the layering and cell types of the presubiculum with their preferential projection profiles would be well placed here in the context of the discussion - e.g. an updated version of Figure 6 of Yoder and Taube, 2011.
